# Remote Sensing Performance Enhancement in Hyperspectral Images

**DOI:** 10.3390/s18113598

**Published:** 2018-10-23

**Authors:** Chiman Kwan

**Affiliations:** Signal Processing, Inc., Rockville, MD 20850, USA; chiman.kwan@signalpro.net; Tel.: +1-240-505-2641

**Keywords:** remote sensing, hyperspectral images, resolution enhancement, pansharpening, spectral resolution, band synthesis

## Abstract

Hyperspectral images with hundreds of spectral bands have been proven to yield high performance in material classification. However, despite intensive advancement in hardware, the spatial resolution is still somewhat low, as compared to that of color and multispectral (MS) imagers. In this paper, we aim at presenting some ideas that may further enhance the performance of some remote sensing applications such as border monitoring and Mars exploration using hyperspectral images. One popular approach to enhancing the spatial resolution of hyperspectral images is pansharpening. We present a brief review of recent image resolution enhancement algorithms, including single super-resolution and multi-image fusion algorithms, for hyperspectral images. Advantages and limitations of the enhancement algorithms are highlighted. Some limitations in the pansharpening process include the availability of high resolution (HR) panchromatic (pan) and/or MS images, the registration of images from multiple sources, the availability of point spread function (PSF), and reliable and consistent image quality assessment. We suggest some proactive ideas to alleviate the above issues in practice. In the event where hyperspectral images are not available, we suggest the use of band synthesis techniques to generate HR hyperspectral images from low resolution (LR) MS images. Several recent interesting applications in border monitoring and Mars exploration using hyperspectral images are presented. Finally, some future directions in this research area are highlighted.

## 1. Introduction

Remote sensing using multispectral (MS) and hyperspectral (HS) images can help fire detection [1], anomaly detection [2,3,4,5,6], chemical agent detection [7], border monitoring [8], target detection [9,10,11], and change detection [12,13,14]. Due to hardware limitation in data storage and scarce bandwidth in data downlink, different imagers have chosen to have different priorities among spatial, spectral, and temporal resolutions. In general, if an imager has a high spatial resolution, then it cannot have a high spectral resolution at the same time and vice versa. For example, Worldview-4 has sub-meter spatial resolution, but has only five bands; Landsat has 11 bands, but has only 30 m resolution; and NASA’s future hyspiri mission has more than 200 bands but with 60 m resolution [15]. Similarly, if an imager has a high temporal resolution, then it cannot have a high spatial resolution or high spectral resolution at the same time. For instance, MODIS has a high temporal resolution (daily), but has 500 m resolution, whereas Landsat has 30 m resolution but a 16-day revisit period. Although NASA’s hyspiri [16] hyperspectral imager can provide global coverage, its spatial resolution of 60 m is still not enough for many applications such as tent detection in refugee camps, car detection in parking lots, etc. It would be useful to improve the spatial resolution of low resolution (LR) hyperspectral images by fusing them with HR color or MS images. Color images are becoming less difficult to obtain nowadays, e.g., Google Map’s color images can achieve 0.5-m resolution. In [17], a new resolution enhancement method was proposed that improves the resolution by injecting information from high resolution color images acquired by other types of imagers, such as satellite or airborne image sensors, to the LR hyperspectral image.

In many remote sensing applications, it will be ideal for images to have high resolution spatially, spectrally, and temporally. In reality, hardware does not allow the above ideal situation to happen and this is where image fusion comes into play. For hyperspectral images, one popular and well-known fusion method is pansharpening. Pansharpening refers to the use of a HR panchromatic (pan) band to sharpen LR MS bands. Many approaches have been proposed in the past two decades. In recent years, new pansharpening approaches have been developed that can utilize HR MS bands for pansharpening (see [18,19,20] and references therein). 

In the spatial resolution enhancement area for hyperspectral images, there are quite a few new developments. Traditionally, single image super-resolution algorithms can be applied. Within this category, there are deep learning, dictionary based methods, etc. Here, we highlight some of the challenges in using single image super-resolution methods. For example, the lack of training images for deep learning based methods necessitates the development of some remote sensing image databases. There are also various fusion/pansharpening methods. We can divide into two categories depending on the availability of PSF. We provide a brief survey of the existing fusion/pansharpening algorithms and highlight some of their advantages and disadvantages.

However, there are some practical issues in pansharpening the hyperspectral images. Many papers in the literature assume some HR pan, color, or MS images are available. This is not the case in general. Collecting HR pan, color, and MS images requires a pro-active approach, which we would like to advocate in this paper. Another issue is related to image registration. There are still some unresolved issues in aligning images from different sources due to different view angles. We highlight some of these problems in the registration process. There are also additional issues related to the availability of point spread function (PSF) and on how to reliably and consistently assess the performance of pansharpened images when ground truth HR hyperspectral images are not available. We offer some suggestions to alleviate the aforementioned issues. 

In the event that no hyperspectral images are available, it is still possible to synthesize hyperspectral images using high resolution MS images. Compared with pansharpening, band synthesis or spectral resolution enhancement is relatively underdeveloped. However, this area is really fascinating and rewarding. We advocate a two-step approach to synthesizing hyperspectral images. First, if HR pan band and low resolution MS bands are available, the MS images are pansharpened first. Second, high performance band synthesis techniques are applied to generate hyperspectral bands from the pansharpened MS images. One key advantage is that once the hyperspectral image cube is generated, many existing algorithms such as anomaly detection, change detection, etc. in the hyperspectral image processing literature can be readily applied. We report some recent studies that show dramatic improvement for object detection using synthetic hyperspectral images.

We believe there are still many research topics in applications using hyperspectral images. One of them is change detection. For example, change detection of the number of tents over time in refugee camps would enable humanitarian agencies to plan the supply chain and prepare in advance for future growth. However, there are still some practical issues such as changes due to natural vs. man-made factors. Another notable one is computational requirements, as hyperspectral images involve a lot of bands.

**Figure 1:**
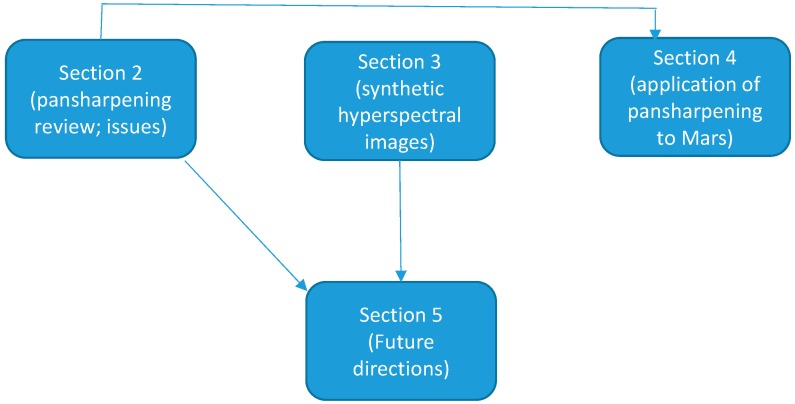
Relationships between the different sections in the revised paper.

In this paper, we briefly review some recent developments in pansharpening in Section 2. These include algorithms for single image super-resolution and pansharpening. The impact of enhanced spatial resolution on target detection and pixel clustering will be illustrated through some practical applications. We also present a few practical issues in pansharpening the hyperspectral images. Some suggestions for alleviating the issues are mentioned. In the event of only HR pan and LR MS images are available, we present some ideas for generating high spatial resolution hyperspectral images in Section 3. An interesting application to border monitoring is included. This paper is by no means a thorough literature survey paper, as we only present some representative algorithms and their applications. In Section 4, we present a recent application in remote sensing in Mars exploration using hyperspectral thermal imagers. Section 5 mentions some future directions in this area. Finally, concluding remarks are included in Section 6. To help readers understand the relationship between different sections, a flow chart is included below in Figure 1.

## 2. Hyperspectral Image Resolution Enhancement Approaches

In this section, we briefly review some resolution enhancement algorithms for HS images. One category is the single image super-resolution approaches. Another category is the image fusion or pansharpening approach. We then discuss some practical issues related to image resolution enhancement.

### 2.1. Single Image Super-Resolution

Here, only a single image is used in improving the spatial resolution of each band in the HS image cube. There are some representative algorithms in this category. The simplest method is the bicubic interpolation, which does not utilize any external information such as point spread function (PSF) [21]. Bicubic interpolation uses 16 neighbors to generate a prediction and the performance is better than bilinear interpolation, which uses only four neighbors. Recently, there are some new developments. One notable development is the algorithm described in [22], which utilizes the PSF to improve the resolution of a single image. The super-resolution algorithm in [23] is based on edge interpolation. There is also a group of methods based on deep learning [24,25]. Vast amounts of training images are needed to train the algorithm. Another group is using dictionary based approach [26,27]. Both the deep learning and dictionary approaches require many training images, which may be difficult to obtain, especially in the remote sensing area. We looked at the open remote sensing website (https://openremotesensing.net/kb/data/) to see if there are some collected remote sensing data that are suitable for deep learning methods. Unfortunately, we could not find any. It would be good for the remote sensing community to build a large image database so that deep learning and dictionary based algorithms can be compared and evaluated.

We would like to emphasize there are many new deep learning based algorithms for enhancing image resolution of images in recent years (see [28,29,30,31,32,33,34,35] and references therein).

It should be noted that single image super-resolution methods can be combined with other fusion methods [17,36]. The idea is to perform a deblurring step by using PSF to the LR hyperspectral images. The deblurred hyperspectral images are then fused with HR color or MS images. 

Now, we would like to use two hyperspectral images to compare a few representative single image super-resolution methods. The two hyperspectral image datasets are: (1) AF data from the Air Force [12]; and (2) AVIRIS data from NASA [37]. The AF image has a size of 267 × 342 × 124, ranging from 0.461 μm to 0.901 μm, meaning that the AF images cover up to the visible and near infrared ranges. The AVIRIS image has a size of 300 × 300 × 213, ranging from 0.38 μm to 2.5 μm. The AVIRIS images cover up to the short-wave infrared (SWIR) range. More details about these images can be found in [17]. 

**Figure 2:**
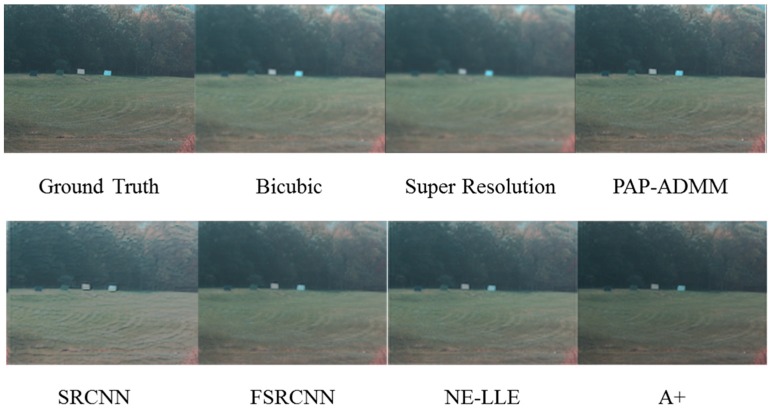
Comparison of seven single image super-resolution algorithms using the AF hyperspectral image in the visible range.

**Figure 3:**
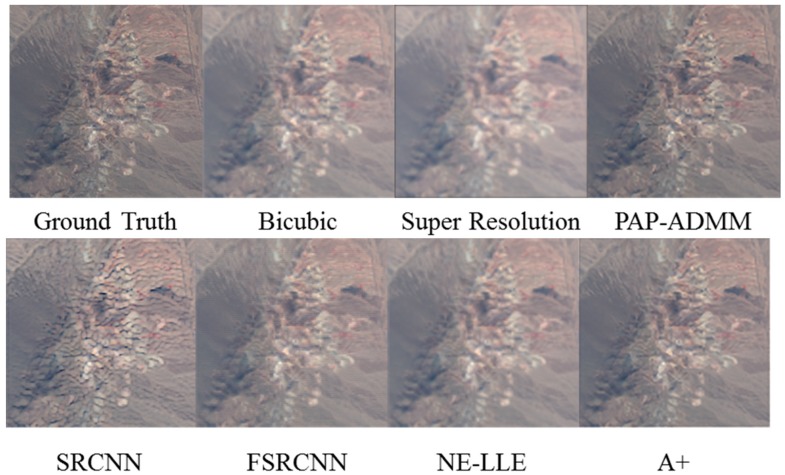
Comparison of seven single image super-resolution algorithms using the NASA AVIRIS data in the visible range.

Figure 2 and Figure 3 compare the results using super-resolution [23], plug-and-play alternating direction multiplier method (PAP-ADMM) [22], deep learning based algorithms (SRCNN [24], FSRCNN [25]), dictionary based algorithms (NE-LLE [27] and A+ [26]), and bicubic interpolation [38]. It can be seen that bicubic and super-resolution methods do not yield much improved resolution as compared to others. SRCNN [24] and NE-LLE [27] tend to have slightly larger color distortion. PAP-ADMM [22] and A+ [26] can strike a balance between color fidelity and spatial resolution. More detailed comparisons in terms of objective metrics and residual values can be seen in [36]. 

### 2.2. Spatial-Spectral Resolution Enhancement: Pansharpening

Pansharpening is an image fusion approach that fuses one high spatial resolution image with another LR MS image. Earlier pansharpening algorithms are limited to images where the panchromatic band overlaps with the MS bands. However, recent advancements have extended the approach to non-overlapping bands [19,20,39,40]. 

#### 2.2.1. Algorithms

In the survey paper by Loncan et al. [19], the categorization of pansharpening algorithms was based on Bayesian, non-Bayesian, component substitution, etc. Here, we take a different viewpoint based on whether PSF is available.

- Group 1: Group 1 methods require knowledge about PSF that causes blur in the LR HS images. Some representative Group 1 methods include coupled nonnegative matrix factorization (CNMF) [41], Bayesian naïve (BN) [42], and Bayesian sparse (BS) [43]. The hybrid color mapping (HCM) based methods [17,36] also belong to this category. Due to the incorporation of PSF, they produce good results in some images.
- Group 2: Unlike Group 1 methods, which require knowledge about the PSF, Group 2 methods only require an HR pan band. As a result, Group 2 performs slightly worse than Group 1 in some cases. This group contains Principal Component Analysis (PCA) [44], Guided Filter PCA (GFPCA) [45], Gram Schmidt (GS) [46], GS Adaptive (GSA) [47], Modulation Transfer Function Generalized Laplacian Pyramid (MTF-GLP) [48], MTF-GLP with High Pass Modulation (MTF-GLP-HPM) [49], Hysure [50,51], and Smoothing Filter-based Intensity Modulation (SFIM) [52], and some others.

In recent years, there are also new deep learning based algorithms [28,29,30,31,32,33,34,35], which normally require thousands and millions of images for training. The lack of remote sensing image databases for training may limit the widespread usage of deep learning based methods in hyperspectral images. 

#### 2.2.2. Visual Performance Comparison 

Similar to Section 2.1, we use the same two datasets to visually compare some of the methods in Group 1 and Group 2. Objective comparisons can be found in [36].

**Figure 4:**
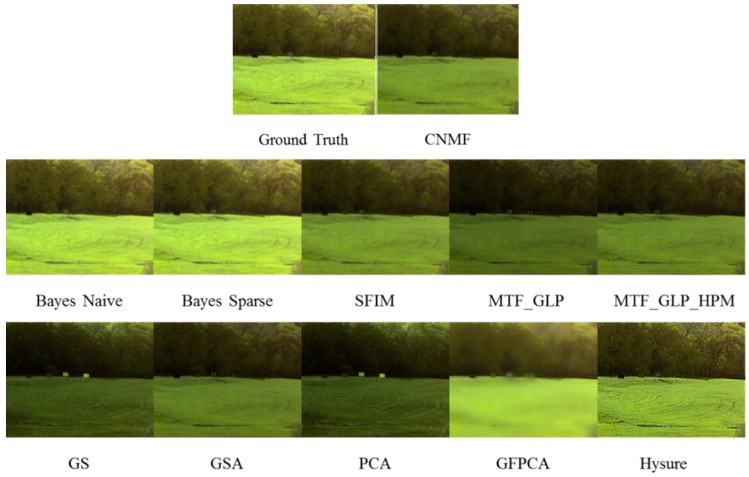
Fused AF images using different methods in the visible near infrared (VNIR) range.

**Figure 5:**
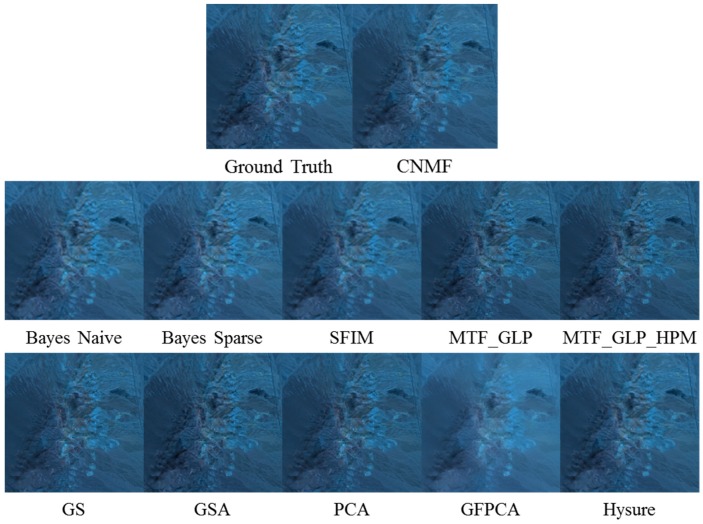
Fused AVIRIS images using different methods in the VNIR range.

In Figure 4, it can be seen that CNMF, SFIM, MTF-GLP, MTF-GLP-HPM, GS, GSA, and PCA have large color distortion, whereas GPPCA has large spatial distortion. Bayes Naïve and Bayes Sparse methods yield close resemblance to the ground truth. In Figure 5, all except GFPCA work well for the AVIRIS data.

#### 2.2.3. Soil Detection Performance Enhancement Using Pansharpened Images

**Figure 6:**
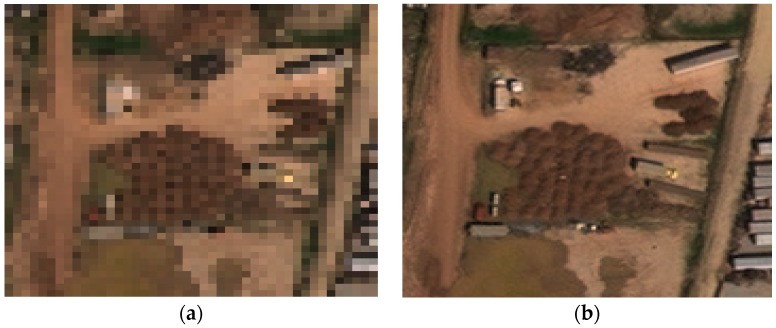

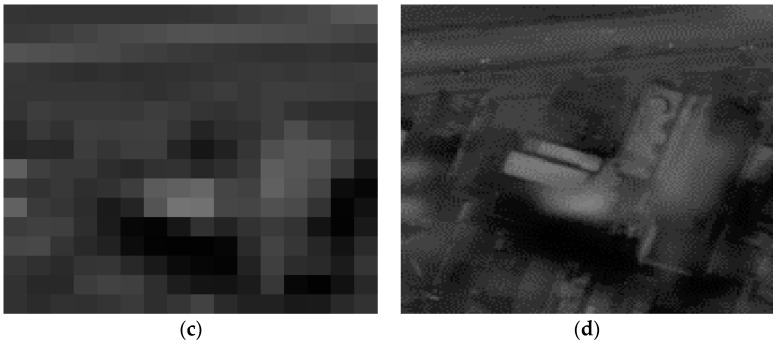
Visual comparison before and after pansharpening for RGB and SWIR images: (**a**) MS RGB band (2 m); (**b**) pansharpened RGB (0.5 m); (**c**) original SWIR image (7.5 m); and (**d**) pansharpened SWIR image (0.5 m).

It is natural to ask the following question: Although pansharpened images can certainly enhance the visual performance, how does the resolution enhancement translate into performance gain in practical remote sensing applications? In previous papers [36,53], pixel clustering application was used to demonstrate that pansharpened images can indeed help improve the clustering performance in hyperspectral images. Moreover, the studies carried out in [39,54] also demonstrated some performance gains when pansharpened images are used for Mars rover image analysis. Here, we include some results on soil detection for illegal tunnel detection. The objective is to use satellite images to detect excavated soil from illegal tunnel digging. Figure 6 shows the enhanced pansharpened images in the multispectral and shortwave infrared (SWIR) ranges. As can be seen in Table 1 for a particular test date, the soil detection performance using joint sparse representation (JSR) [8], kernel JSR [8], matched subspace detector (MSD) [55], kernel MSD (KerMSD) [56], Support Vector Machine (SVM) [57], and pixel-wise sparse representation (SR) [58] methods have been improved quite a lot after pansharpening. More details can be found in [8].

#### 2.2.4. Pixel Clustering Enhancement

To further demonstrate that the enhanced spatial resolution after pansharpening can also help target classification, we provide some results related to pixel clustering using different pansharpening algorithms. Pixel clustering was not performed in any of the competitive approaches [25,26,41,42,43,44,45,46,47,48,49,50,51,52].

**Figure 7:**
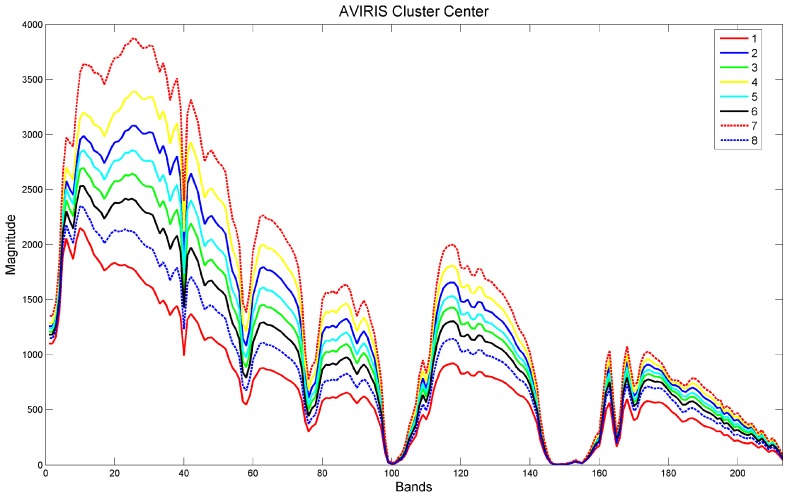
Spectral signatures of the eight cluster centers.

We emphasize the following points:

- This study is not for land cover classification. In land cover classification, it is normally required to have reflectance signatures of different land covers and the raw radiance images need to be atmospherically compensated to eliminate atmospheric effects.
- Because our goal is for pixel clustering, we worked directly in the radiance domain without any atmospheric compensation. The clustering was done using the k-means algorithm. The number of clusters selected was eight in the AVIRIS datasets. Although other numbers could be chosen, we felt that eight clusters would adequately represent the variation of pixels in these images. The eight signatures or cluster means of AVIRIS dataset are shown in Figure 7, respectively. It can be seen that the clusters are quite distinct.
- Moreover, since our focus is on pixel clustering performance of different pansharpening algorithms, the physical meaning or type of material in each cluster is not the concern of our study.
- Other classification and clustering could be used [59,60] for pixel clustering. We used the simplest method. A pixel is considered to belong to a particular cluster if its distance to that cluster center is the shortest. Here, distance is defined as the Euclidean distance between two pixel vectors. The main reason is that some of the cluster means in Figure 7 have similar spectral shapes. If we use spectral angle difference, then there will be many incorrect results.

**Figure 8:**
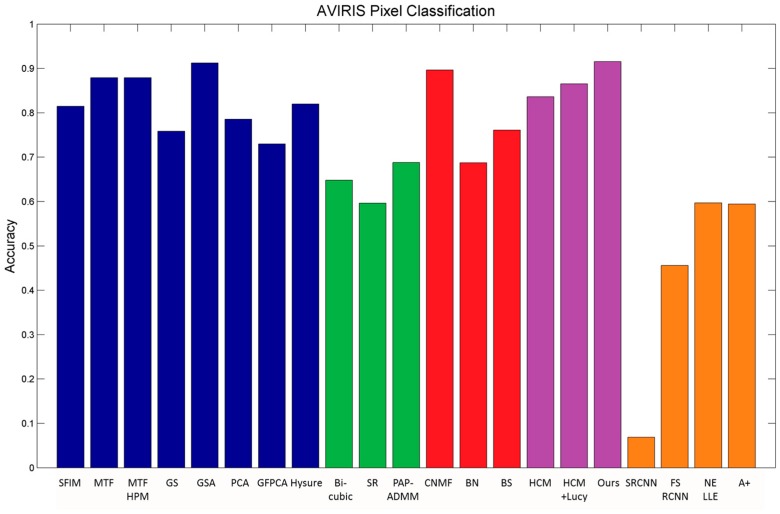
Clustering accuracy using different algorithms for the AVIRIS dataset. Red: Group 1 methods; Blue: Group 2 methods; Green: Single image super-resolution methods; Purple: HCM methods; Orange: Deep learning and dictionary based methods.

It is our belief that, if a pansharpening algorithm can preserve the spatial and spectral integrity in terms of peak-signal-to-noise ratio (PSNR), correlation coefficient (CC), and spectral angle mapper (SAM), and can also achieve a high pixel clustering accuracy, it should be regarded as a high performing algorithm. Figure 8 shows the pixel clustering accuracy of many algorithms, including single image super-resolution, Group 1 and Group 2 methods, HCM based methods, and deep learning and dictionary based methods. The performance varies a lot among the different methods. More results can be found in [36]. We also mention that, since no single method can perform well under all conditions, it is necessary to have more diverse methods so that researchers can best select the most appropriate algorithms for their applications.

### 2.3. Practical Issues in Pansharpening Hyperspectral Images

Pansharpening of hyperspectral images requires high resolution pan, color, or MS images. In many papers ([17,18,19,20,36] and references therein), researchers assume those HR images are available. In practice, this is not the case. We discuss some other practical issues in pansharpening. We also attempt to offer some suggestions that may alleviate these issues.

#### 2.3.1. Availability of High Resolution Data

When we started this pansharpening/fusion effort a few years ago, we sought some HR color images and LR hyperspectral images that have the same time and location. However, it was difficult to find matched images for several reasons. First, the high resolution color images mainly come from Digital Globe’s images, which do not have regular revisit times over the same location. For example, we have been investigating border monitoring using Worldview images and have looked at the Millerovo airport near the Russian–Ukraine border. We found that there are only a few images over a two-year period in 2014 and 2015. The NASA Cuprite data were collected on 19 June 1997 and there was no corresponding high resolution color image near that date. We also looked at the well-known site containing hyperspectral image data (http://lesun.weebly.com/hyperspectral-data-set.html). The HYDICE image of the Washington DC Mall area was collected on 23 August 1995 and the AVIRIS image Indian Pine dataset was collected on 12 June 1992. The high resolution commercial color images are only available since 2010 (See https://www.digitalglobe.com/resources/satellite-information). 

In recent years, Planet (a commercial satellite company) has launched many cubesats to cover the Earth globally. The revisit times may be more often now. We believe a more proactive approach to hyperspectral data collection may be as follows. Before one collects hyperspectral data for a given location, it would be better to know the revisit dates/times of the high resolution imagers (Planet, Worldview, and possibly others) for that location and then collect data for the same day. This may allow simultaneous collection of high resolution color images with low resolution hyperspectral images.

#### 2.3.2. Registration Issues

**Figure 9:**
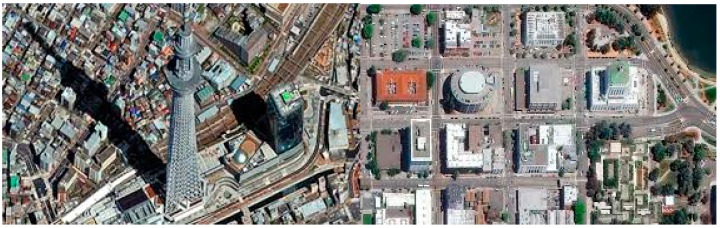
Exemplar off-nadir high resolution images from WV-2.

**Figure 10:**
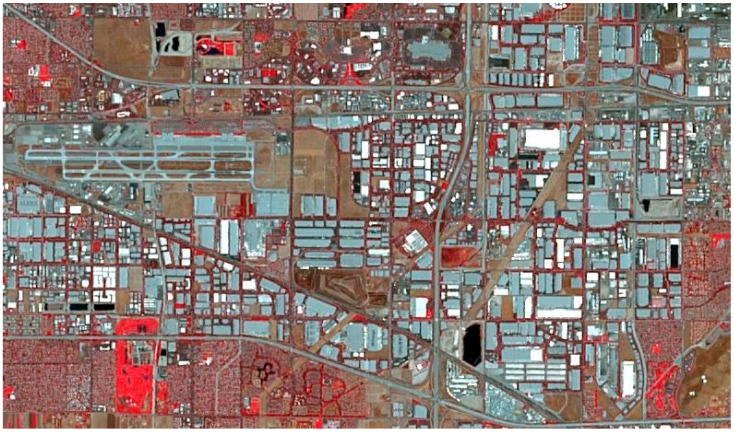
Landsat images are collected nadir. Sides of buildings cannot be seen.

Even if we find two matched datasets (one HR color/MS and one LR hyperspectral) that have the same date and location, there are some practical issues. One issue is the alignment, which requires subpixel accuracy. It is well-known that the Worldview images are collected off-nadir. See some representative images in Figure 9. One can see that the building sides can be seen, meaning that the images are collected off-nadir. It is not straightforward to apply feature based registration algorithms to align these sort of images. Recently, we tried to align Worldview (off-nadir) images with Landsat (nadir). See one exemplar Landsat image in Figure 10. We had to resort to manual alignment by selecting some ground control points because features from building corners cannot be used. This is because buildings in Worldview images are mostly slanted due to off-nadir data collection. To the best of our knowledge, automated image alignment between Worldview images and other images such as Landsat still needs more research. We suggest that an automated approach to locating ground feature points (road intersections) will likely solve the registration problem.

#### 2.3.3. Lack of PSF Information

Some pansharpening algorithms [17,36,41,42,43] require the PSF information. Normally, during the sensor calibration process, each sensor manufacturer uses a calibration pattern to determine the PSF. The frequency domain counterpart of PSF is known as modulation transfer function (MTF). However, the PSF or MTF is usually not made known to the public. Thus, in theory, each imager should have a PSF. In practical applications where PSF is not publicly available, there are some blind deblurring algorithms discussing ways to estimate the PSF. For example, Prof. Jiaya Jia’s group (http://jiaya.me/deblurring.htm) has developed algorithms to estimate PSF. Based on our experience in blind deblurring, this area is still under development and more research is needed.

#### 2.3.4. Image Quality Assessment

To assess the quality of pansharpened images, there are two approaches. One is to apply the Wald’s protocol [19], which assumes the HR hyperspectral images are available. The other is the full resolution assessment approach [19]. A performance metric known as Quality with No Reference (QNR) has been used. However, if a method performs well using Wald’s protocol, it does not mean that it will also have high performance using full resolution assessment (e.g., QNR). This can be clearly seen in Table XIII of Vivone et al.’s 2015 paper [20]. In that table, one can see that the MTF-GLP-HPM-PP and AWLP methods are categorized to have high performance by using Wald’s protocol, but are considered to have poor performance by using QNR. There are several other inconsistencies in that table. For example, PRACS [61] has medium performance using Wald’s protocol, but was considered as high performingusing QNR. In addition, in Table XII of [20], the ranking of different methods is very different from those in Table XIII because the results in the two tables were generated using two different datasets. 

In [18], a generalized QNR (GQNR) algorithm was proposed for evaluating pansharpened images using Worldview 3 (WV-3) data. The GQNR results agreed well with subjective evaluations. We believe GQNR may be suitable for assessing pansharpening algorithms in hyperspectral images. In any event, more research needs to be done in this area.

## 3. Performance Enhancement Using Synthetic Hyperspectral Images

As seen in earlier sections, if HR pan, color, or MS images are available, then pansharpening can indeed enhance the resolution of hyperspectral images. Consequently, both visual, pixel clustering, soil detection performance, etc. can then be improved. In some applications, however, we have access to only HR pan and LR MS images. Could we synthesize some hyperspectral images using those images? In early 2000, Prof. C.-I. Chang’s group started the effort on spectral band synthesis [62]. Since then, there have been some newer and better algorithms such as the Extended Morphological Attribute Profiles (EMAP) algorithm [63,64,65,66,67]. 

Here, we present a two-step approach to band synthesis assuming that we have a HR pan and LR visible near infrared (VNIR) and shortwave infrared (SWIR) images. This is the case for Worldview-3 images where there is a HR pan band with 0.5 m resolution, 8 VNIR bands with 2 m resolution, and 8 SWIR bands with 7.5 m resolution. First, we apply the HR pan band to enhance the resolution of both the VNIR and SWIR bands. Second, we propose to apply band synthesis to generate synthetic HR hyperspectral images. We describe the above ideas and show some interesting and excellent soil detection performance below.

### 3.1. Enhancing VNIR and SWIR Bands Using the HR Pan Band

**Figure 11:**
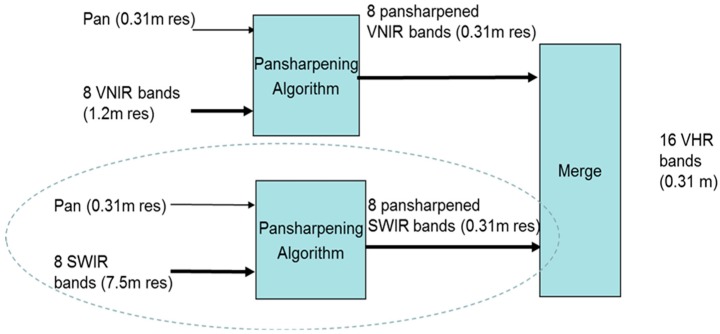
Approach 1: Parallel one-step approach to generating HR VNIR and SWIR bands.

**Figure 12:**
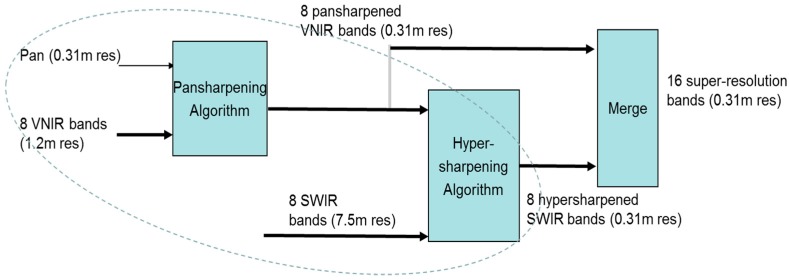
Approach 2: Sequential Fusion.

**Figure 13:**
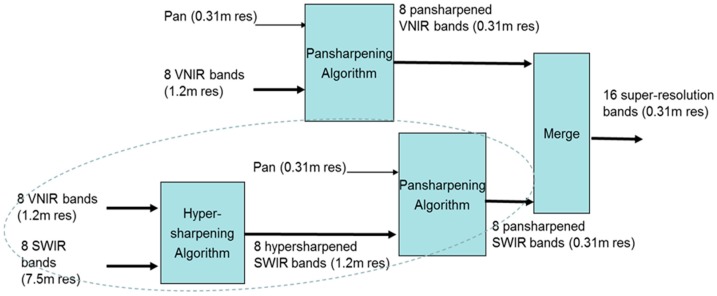
Approach 3: Sequential-parallel fusion of pan, VNIR, and SWIR bands.

**Figure 14:**
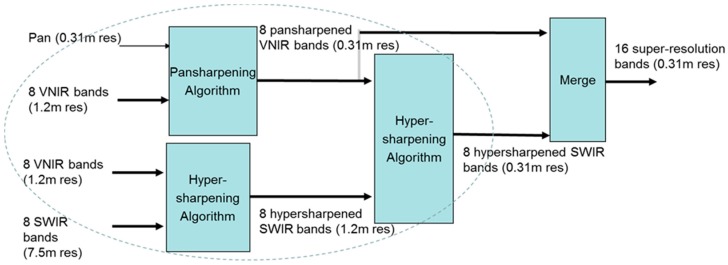
Approach 4: Parallel-sequential fusion approach to generating HR VNIR and SWIR bands.

When HR pan, medium resolution VNIR, and LR SWIR bands are all available, conventional approaches will simply apply pansharpening to the VNIR and SWIR bands separately and directly. In a recent research [18,68], three new additional approaches are proposed to enhance the SWIR bands. For completeness, we include the block diagrams of these four approaches below. Figure 11 illustrates Approach 1, which is the conventional approach. The HR pan is used to pansharpen the VNIR and SWIR bands separately. Figure 12 shows a sequential fusion approach where the HR pan is used to pansharpen the VNIR bands first. The pansharpened VNIR bands are then used to pansharpen the SWIR bands. Figure 13 shows one unconventional approach where the VNIR bands are used to pansharpen the SWIR bands to the same resolution of the VNIR bands. Then, the HR pan band is applied to pansharpen the pansharpened SWIR bands to the same resolution of pan band. Figure 14 shows a more complicated approach which begins with a parallel pansharpening step, followed by another sequential fusion step. In terms of computational complexity, Approaches 3 and 4 require more computations.

Experimental results in [18] show that Approaches 3 and 4 yielded better performance than the rest. Between Approaches 3 and 4, Approach 3 is slightly better.

### 3.2. Synthetic Hyperspectral Bands for Enhanced Soil Detection

After we generate HR MS bands using methods described in Section 4.1, we can proceed to the second step of synthesizing hyperspectral bands using the HR MS bands. 

Since the emergence of hyperspectral imagers, material classification accuracy was found to be much better than that of MS images. In [62], the authors investigated ways to synthesize spectral bands out of the MS bands. The aim was to create a high number of bands so that target classification performance can be improved. Recently, some new algorithms known as Extended Morphological Attribute Profiles (EMAP) were developed [63]. Source codes can be requested from the authors of [63]. These new algorithms turned out to significantly improve the classification performance [65,66,67,69,70].

EMAP [63] is an extended idea of attribute profile (AP), a method that has recently been presented as an efficient tool for spectral-spatial analysis of remote sensing images [64]. APs provide a multi-level characterization of an image obtained by applying a sequence of morphological attribute filters to model different kinds of structural information on a single-band (or grayscale) image. These attribute filters can be morphological operators (so-called features) such as thinning or thickening operators that process an image by merging its connected pixels. APs using different types of attribute features on different threshold levels can be stacked together, generating Extended Multi-Attribute Profiles (EMAPs) [63].

Mathematically, given an input grayscale image f and a sequence of threshold levels $\left\{T_{1},\;T_{2},\;\ldots \;T_{n}\right\}$, the AP of f is obtained by applying a sequence of thinning and thickening attribute transformations to every pixel in f:

(1) $$AP\left(f\right)=\{\phi_{1}\left(f\right),\;\phi_{2}\left(f\right),\;\ldots \;\phi_{n}\left(f\right),f,\;\gamma_{1}\left(f\right),\;\gamma_{2}\left(f\right),\;\ldots \;\gamma_{n}\left(f\right)\}$$

where $\phi_{i}$ and $\gamma_{i}\;\left(i=1,\;2,\;\ldots n\right)$ are the thickening and thinning operators at threshold $T_{i}$, respectively. The EMAP of f is then acquired by stacking two or more APs using any feature reduction technique on multispectral/hyperspectral images such as purely geometric attributes (e.g., area, length of the perimeter, image moments, and shape factors) or textural attributes (e.g., range, standard deviation, and entropy) [63].

(2) $$EMAP\left(f\right)=\left\{AP_{1}\left(f\right),\;AP_{2}\left(f\right)\;\ldots \;AP_{m}\left(f\right)\right\}$$

As can be seen from the above, the EMAP process generates some “virtual features” through nonlinear morphological filters (thinning and thickening) and some attribute extraction steps (diagonal, area, etc.). Those nonlinear operations create some perturbations to the original bands and eventually enhance the detection performance.

**Figure 15:**
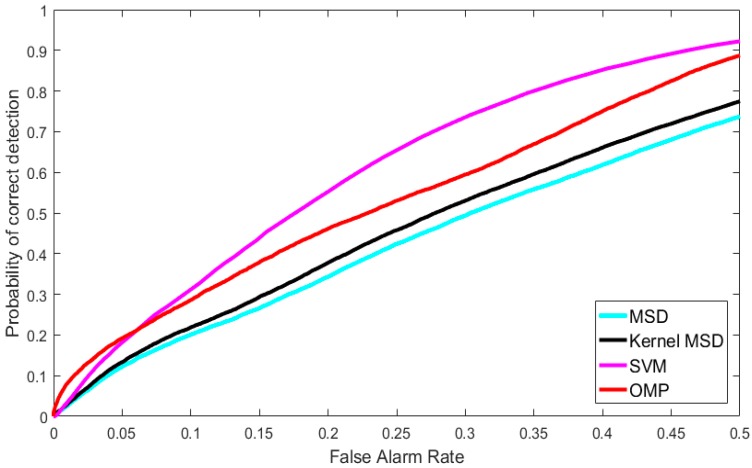
Performance of using color images for soil detection.

**Figure 16:**
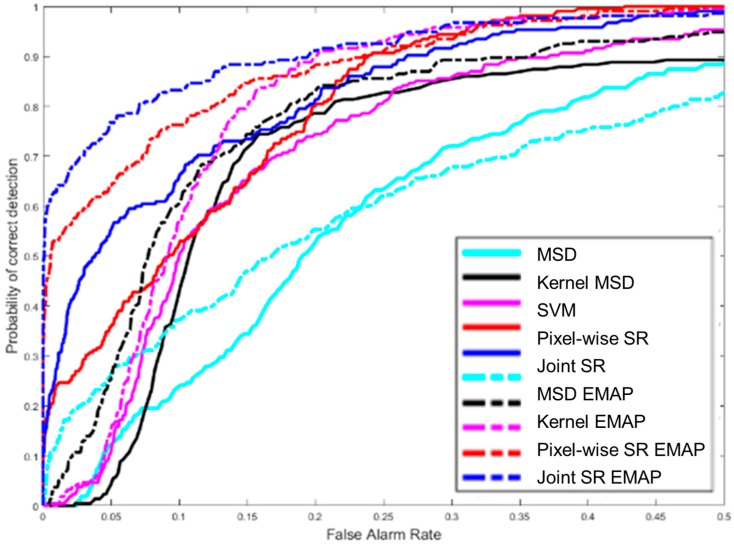
Detection results using synthetic bands.

Now, we demonstrate the advantages of using synthetic hyperspectral images for soil detection in border monitoring. We have eight MS bands of Worldview-3 images at 2 m resolution. We applied EMAP to generate 80 synthetic bands. It should be noted that no pansharpening was done to the MS images in this study, as our focus was on demonstrating the performance gain using synthetic bands. The 80 synthetic bands and eight MS bands were then combined to carry out soil detection. Figure 15 shows the performance of using color images for soil detection. The performance was not good. None of the detection methods (MSD [55], kernel MSD [56], SVM [57], and Orthogonal Matching Pursuit (OMP) or (JSR) [8]) worked well. OMP or JSR is a sparsity based approach that minimizes the number of sparse coefficients in the sparse representation. In contrast, Figure 16 shows results of using synthetic hyperspectral images with 88 bands for soil detection. Several detection methods were used: MSD, kernel MSD, SVM, and sparsity based approaches (pixel-wise SR and joint SR). The performance gain by using synthetic bands is dramatic. For example, at 5% false alarm rate (FAR), the correct detection rate has been improved by close to 20% using the joint sparsity method. In general, using synthetic bands improves the performance of every method. More details can be found in [69].

## 4. Application to Surface Characterization of Mars Using Hyperspectral Data

In earlier sections, we present resolution enhancement algorithms and their applications to border monitoring and pixel classification applications related to terrestrial applications. Here, we present one interesting and unique remote sensing application for Mars surface characterization that utilize techniques (pansharpening) mentioned earlier. Only partial results are shown here, as a very detailed paper [71] has been submitted to a conference.

### 4.1. THEMIS and TES Fusion

#### 4.1.1. THEMIS and TES Imagers and Data

Thermal Emission Imaging System (THEMIS) with a spatial resolution of 100 m has 10 infrared bands between 6 and 15 microns (centered at: 6.78, 6.78, 7.93, 8.56, 9.35, 10.21, 11.04, 11.79, 12.57, and 14.88 microns). The last band is noisy and the first two bands are actually the same. Therefore, we have only eight THEMIS bands.

Thermal Emission Spectrometer (TES) with a resolution of 3 km has 143 bands between 5 and 50 microns. To minimize the impact of Mars’ atmosphere, atmospheric compensation (AC) is done to the TES data. The end-product is 73-band AC data. We used the Java Mission-planning and Analysis for Remote Sensing (JMARS) tool [72,73] to retrieve the THEMIS and TES data. In addition to the above, there are multispectral imagers (Mastcam) [2,54] and laser induced breakdown spectrometer (LIBS) [74], and Alpha Particle X-Ray Spectrometer (APXS) onboard the Mars rover Curiosity [75].

#### 4.1.2. Generation of Atmospherically Compensated THEMIS Data

**Figure 17:**
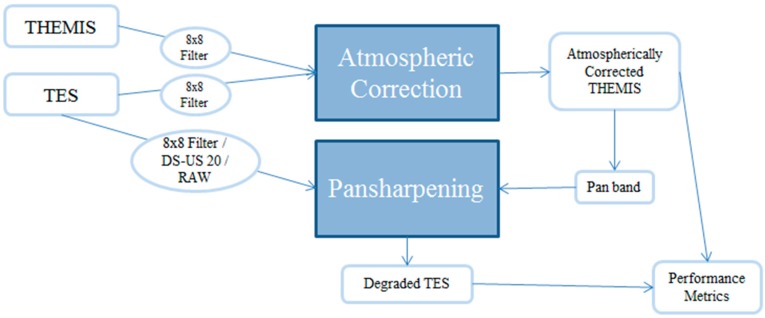
Diagram of THEMIS/TES fusion process.

Figure 17 summarizes the fusion process. The first critical step is the atmospheric compensation process for the THEMIS data. Due to dust, carbon dioxide, and small amount of water vapor in the Mars atmosphere, the raw THEMIS data need some atmospheric compensation. The 73-band atmospherically compensated TES data are used to help create an atmospheric component library which, when applied to the THEMIS image, results in an Atmospherically Corrected (AC) THEMIS image. Though there are several combinations of filtering options for the THEMIS and TES images at this stage, our research is focused on the case in which both the THEMIS and TES images are processed by an 8 × 8 filter before entering the atmospheric correction phase.

Once the AC THEMIS dataset is complete, it is used to pansharpen the 73-band TES image. A panchromatic (pan) band is created by averaging bands 4–9 of the AC THEMIS data. To measure the effectiveness of the selected pansharpening algorithm, degraded TES bands [76] are generated from the pansharpened bands. The resulting degraded TES bands are subsequently compared against the AC THEMIS bands to compute the performance metrics. The result of this process is an image with high spatial resolution and high spectral resolution supported by objective numerical comparison.

### 4.2. Pansharpening Results

**Figure 18:**
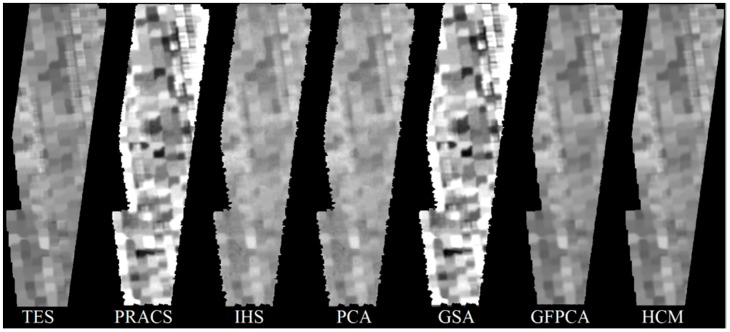
Original TES band 42 and the pansharpened Images across pansharpening algorithms.

Six pansharpening algorithms were evaluated: partial replaced adaptive component substitution (PRACS) [61], intensity hue saturation (IHS) [77], principal component analysis (PCA) [44], Gram–Schmidt Adaptive (GSA) [47], and Guided Filter PCA (GFPCA) [45]. In addition to these five algorithms, we also included our own: hybrid color mapping (HCM) [18,78,79]. Figure 18 shows the pansharpened results of different algorithms. Visual analyses, corroborated by the performance metrics in Table 2, assert HCM, IHS and PCA as the superior pansharpening algorithms in this application. The performance metrics employed are as follows: root mean square error (RMSE), peak signal-to-noise ratio (PSNR), similarity angle metric (SAM), erreur relative globale adimensionnelle de synthèse (ERGAS) and cross-correlation coefficient (CC).

The use of variably-sized spectrally uniform areas (SUAs) [76] has a substantial effect on the performance metrics used to judge the quality of our pansharpened images. Visual inspection of the 1 × 1 SUA images reveals the aforementioned over-compensation during the AC process, resulting in images that are largely indistinguishable from the original TES images. This new AC process produces images that display a clear blend of the THEMIS spatial information and the TES spectral information. 

### 4.3. Mineral Abundance Estimation

To extract the mineral contributions to a given pixel’s emissivity, we must perform spectral unmixing. We do so using the Non-negatively Constrained Least Squares (NCLS) [7] algorithm. This algorithm takes a pixel and a spectral library as its inputs and returns the contribution percentage that each library entry makes to the spectral shape of the image. The spectral library used for this unmixing is presented in Rogers (2008) [80].

**Figure 19:**
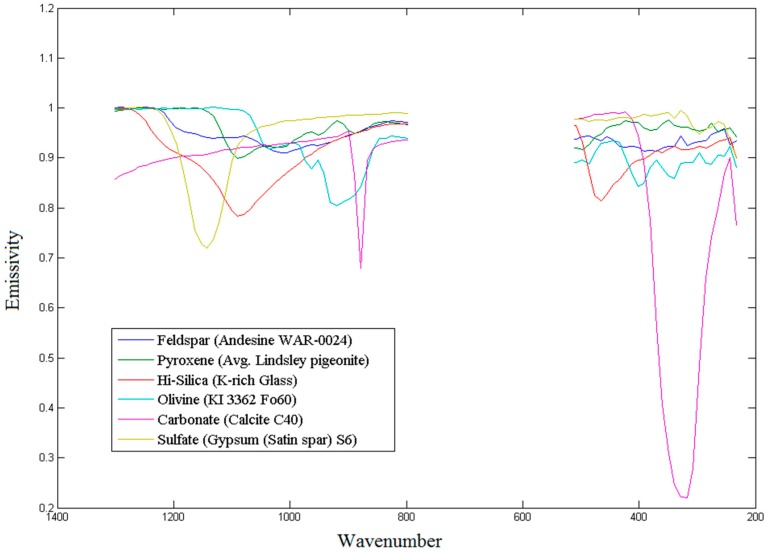
Spectral shapes of representative minerals for mineral groups included in the surface model.

To determine the contribution each mineral group makes to the composition of the bedrock, the individual contributions—the output of the NCLS algorithm—are summed alongside the other members of their groups [81] to produce mineral group totals. Table 3 shows a list of minerals and Figure 19 shows a few representative mineral signatures. For our purposes, contributions below 1% are considered unreliable and are, therefore, not included in the presented totals.

**Figure 20:**
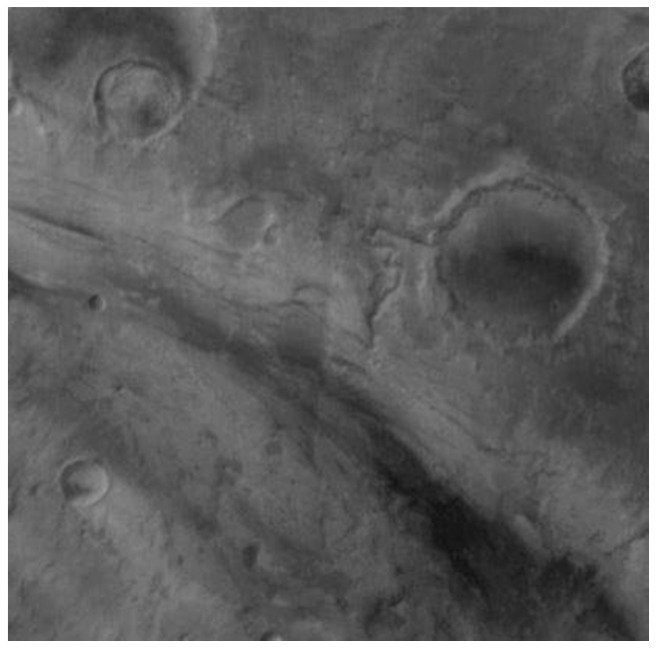
Area of study in the visible range [82].

Figure 20 shows the area of study, which is a section of the Ares Vallis bedrock chosen for its mineralogical heterogeneity [80]. Here, the rock walls contain a layer enriched by Olivine—an indicator of possible flooding, magmatic, or glacial activities. This region and its exposed bedrock have drawn the attention of a fair amount of research in the past, motivating our investigations not only by the area’s curious nature but by the prospect of comparative studies that build off of previously published work.

Our first motivation for this comparative study was to analyze the products of our atmospheric correction algorithm. Similarities between our unmixing results and the figures presented in Rogers [80] demonstrate that our High-Order algorithm is generally consistent with published results. When this has been established, we can proceed to our second motivation: the pansharpening phase. With the knowledge that our AC process is valid, we can pansharpen the TES data using THEMIS images and see what this blended data product reveals about the surface of Mars.

Preliminary results demonstrate that our AC algorithm produces mineral contribution levels which are, in fact, similar to those presented in Rogers [80]. Moreover, the NCLS algorithm produced results that are slightly different when processing the pansharpened images; a finding which indicates that there may be new knowledge to be gained through the fusion of TES and THEMIS products.

Coordinates for the areas used to model the colored units are listed below in Table 4; these coordinates are best-effort matches of the selections made in Rogers [80]. However, due to missing data, a perfect recreation is not possible at the moment (this is particularly pertinent for the Green unit, which cannot be reasonably isolated in I07815026 due to absences in the TES data). Furthermore, the selected areas in Rogers [80] all have an area of at least 90 pixels, whereas our selections are smaller. Instead of aiming to meet a 90 pixel minimum, our selections have an area of 81 pixels (9 × 9) to maximize color purity.

**Figure 21:**
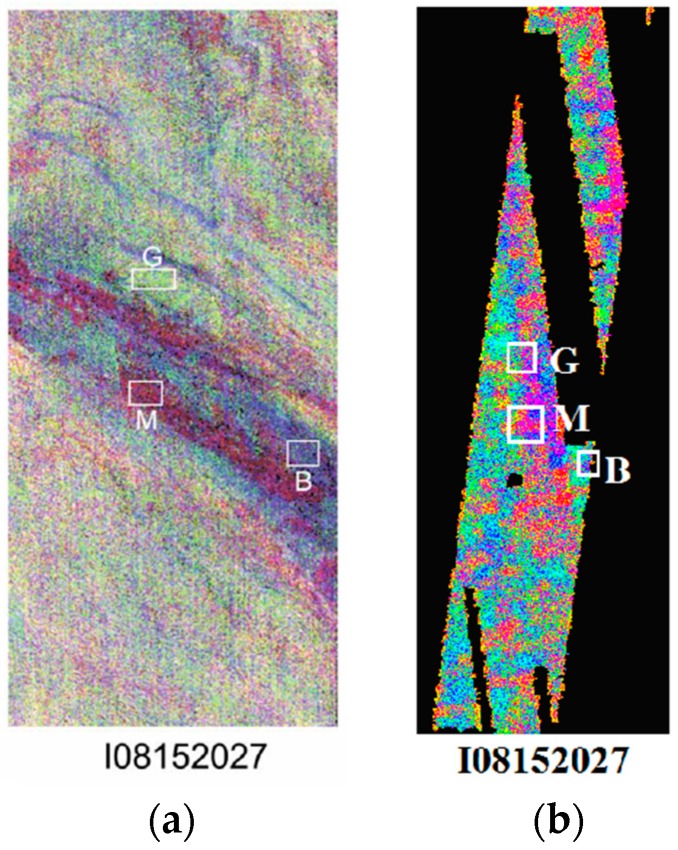
Areas of interest-Decorrelation Stretch of bands 5, 7, and 8 as presented in Rogers [80] (**a**). Decorrelation Stretches of pansharpened area of study images (**b**).

To compare our results to the Rogers dataset, we performed a DCS in MATLAB with the same band orientation (bands 5, 7, and 8 as red, green, and blue, respectively). The THEMIS data have been masked to exclude areas in which the TES image is missing data. The THEMIS images used for this decorrelation stretch are not atmospherically corrected, unlike the Rogers images, due to our investigation into the atmospheric compensation process. The DCS images are shown in Figure 21.

Our studies have provided us with mixed results, as shown in Table 5. See [71] for more details. It seems as though pansharpening the images causes some numbers to move further from those in Rogers, and some to draw closer. For instance, the Magenta unit Feldspar average is drawn 1% higher by pansharpening, while the Blue unit Pyroxene average is drawn 3% closer to the published results by the selfsame process. As we do not have a ground truth in this scenario, it is not possible to say that our results are objectively better or worse. Instead, we present these findings both as new possibilities for the surface of Mars and as verification that the pansharpening process brings a reasonable, yet novel perspective to the mineral characterization endeavor.

**Figure 22:**
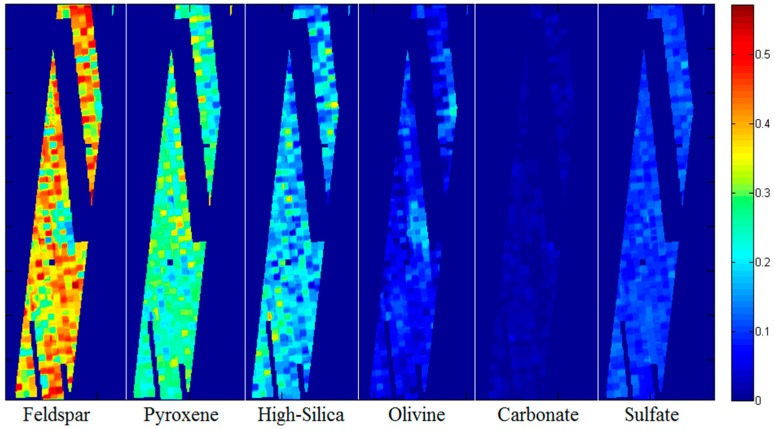
Contribution Maps for I08152027; Color bar represents contribution level. Each image represents the sum of all minerals in the respective group.

Figure 22 shows the abundance maps of different minerals. It can be seen that the area has high concentration of Feldspar, followed by Pyroxene, and silica. The concentration of Feldspar can vary greatly in different locations.

## 5. Future Directions

Despite intensive research in hyperspectral image processing in recent years, there are still some difficult research problems in this area. Here, we list a few of them: 

● Build databases for high and low resolution images collected at the same location and time

To improve the spatial resolution of hyperspectral images by software, it is necessary to first collect high resolution color/MS images simultaneously with the LR hyperspectral images. We recommend that, before one starts collecting hyperspectral data, it is better to know when the HR color/MS imagers will fly over the same area. The collected data will serve two purposes. First, the data will allow pansharpening to be performed. Second, the collected data will also help build some deep learning/dictionary based models for single-image super-resolution.

● Enhance registration algorithms for images from different sources

Since images from different sources may have different times of collection, sun angles, view angles, etc., it is critical to align the images before pansharpening can take place. We think that an automated process of selecting the ground control points (GCP) will be critical in the alignment process. New research is still needed in this area.

● Improve estimation of PSF

Some pansharpening algorithms [36,37,38,39,40,41,42,43] require the PSF to be available. In practice, the PSF may not be available to the public. Some blurring kernel or PSF estimation algorithms do exist. However, based on our evaluations [21], the estimation performance is still not satisfactory. More research is still needed in the near future.

● Further development of band synthesis techniques

Based on our recent investigations, we found that spectral band expansion idea may not always work. In tent detection in refugee camps and soil detection for border monitoring, we saw dramatic improvement. However, for airplane detection using Worldview images, we did not see much improvement. This means that we need to come up with consistent methods that can make the band expansion idea work for all cases.

● Change detection using fused images

Here, fused images are images created by blending HR images with LR images. One well-known example is the fusion of Landsat and MODIS where MODIS has low resolution of 500 m but has daily revisit, and Landsat is just the opposite. More details can be found in [83,84]. Another fusion study is to improve the temporal resolution of Worldview images by fusing them with Planet images [85]. Recently, our team performed a fusion study for Landsat and Worldview images [86]. The goal was to increase the temporal resolution of Worldview images with help from Landsat images. Once the fused images are available, we can perform more frequent change detection for a given area. However, the change detection performance is limited by the performance of the fusion. More research is needed here.

● Computational efficiency enhancement

Due to the large number of bands, hyperspectral image processing is more computationally demanding than that of MS images. Researchers have found efficient ways to speed up the computations. One approach is to apply principal component analysis (PCA) to reduce the number of bands and then some additional tasks such as change detection can be performed. In target recognition area, one practical approach is to work directly in the radiance domain [87,88], as only a handful of target signatures need to be converted from reflectance to radiance. In anomaly detection, there are fast detectors based on random sampling of background pixels [89], clustering of background pixels [12], progressive line scanning [90], and recursive implementation [91].

● Pansharpening performance assessment

Earlier, we mentioned a full resolution approach [18] that was proven to be quite useful for assessing pansharpened Worldview images. More research is still needed to further demonstrate its performance in hyperspectral images.

## 6. Conclusions

Hyperspectral images have been proven to be very useful for target detection and classification. However, one serious limitation is the spatial resolution. In this paper, we review some of the practical issues in enhancing the resolution of hyperspectral images. We also attempt to offer some remedies for alleviating those issues. We then present a brief review of the resolution enhancement algorithms, including single image super-resolution and pansharpening algorithms. Some interesting and practical applications such as border monitoring and pixel clustering are included to demonstrate that enhanced resolution images can help improve the visual and classification performance. Moreover, in the event that only MS images are available, we present some techniques that can synthesize hyperspectral images from the MS images. A recent application on Mars surface characterization using hyperspectral images is also included. Finally, we mention a few future research directions in this area.

## Figures and Tables

**Table 1:** Comparison of detection accuracies for test date 19 March 2010 with false alarm rates (FAR) of 5% and 10%. Bold number highlight best performing methods.

	FAR = 5%		FAR = 10%	
	MS Resolution	Pansharpening	MS Resolution	Pansharpening
MSD	0.46	8.95	23.25	71.45
KerMSD	18.60	28.98	58.13	78.58
SVM	23.72	35.95	42.79	63.20
Pixel-wise SR	53.48	62.77	76.70	86.14
JSR	63.72	64.32	73.49	77.53
KerJSR	**68.37**	**74.09**	**76.28**	**90.36**

**Table 2:** Performance Metrics for Filtered THEMIS + Filtered TES (TES Filtered during Pansharpening).

	RMSE	PSNR	SAM	ERGAS	CC
PRACS	0.030934	30.191335	0.087938	0.250943	0.999555
IHS	0.007594	42.390412	0.069292	0.061094	0.999997
PCA	0.007640	42.338531	0.069362	0.061441	0.999997
GSA	0.033318	29.546523	0.069240	0.268104	0.999525
GFPCA	0.012954	37.751836	0.083463	0.106650	0.999878
HCM	0.002573	51.790357	0.067877	0.020901	0.999995

**Table 3:** Mineral library used for modeling Mars’ surface [80].

Quartz BUR-4120	Shocked An 27.0 GPa	Forsterite BUR-3720A	SiO_2_ Glass
Microcline BUR-3460	Shocked An 38.2 GPa	Fayalite WAR-RGFAY01	02-011 Opal A
Albite WAR-0235	Shocked An 56.3 GPa	KI 3362 Fo60	aluminous opal scale-pellet
Oligoclase BUR-060D	Bronzite NMNH-93527	KI 3115 Fo68	Crystalline heulandite (zeo)
Andesine WAR-0024	Enstatite HS-9.4B	KI 3373 Fo35	Crystalline stilbite (zeo)
Labradorite BUR-3080A	Hypersthene NMNH-B18247	KI 3008 Fo10	Average Martian Hematite
Bytownite WAR-1384	Avg. Lindsley pigeonite	Imt-1 < 0.2 microns	Anhydrite S9
Anorthite BUR-340	Diopside WAR-6474	Montmorillonite (Ca) STx-1	Gypsum (Satin spar) S6
Shocked An 17 GPa	Augite NMNH-9780	Saponite < 0.2 microns	Kieserite
Shocked An 21 GPa	Augite NMHN-122302	Swy-1 < 0.2 microns	Calcite C40
Shocked An 25.5 GPa	Hedenbergite (Manganoan) DSM-HED01	K-rich Glass	Dolomite C20

**Table 4:** Coordinates of the cropped areas used to represent the DCS color units. Each cropped area is a 9-by-9 pixel square whose upper left corner is located at the specified coordinate. See boxes in Figure 21b.

	Magenta	Blue	Green
I08152027	1222, 1011	1379, 1090	1261, 916

**Table 5:** Mineral group contribution means across three image types. “*Green*” unit averages are obtained only from images I08539014 and I08152027, as a Green unit could not be reliably isolated in I07815026.

	Raw TES			Pansharpened TES			Rogers (2005)		
	Magenta	Blue	Green*	Magenta	Blue	Green*	Magenta	Blue	Green
Feldspar	21%	38%	26%	22%	35%	26%	10%	30%	20%
Pyroxene	29%	26%	25%	28%	24%	25%	40%	25%	15%
High-Silica	13%	17%	21%	16%	15%	21%	10%	25%	35%
Olivine	15%	4.7%	13%	15%	4%	12%	15%	5%	10%
Carbonate	2%	2%	1%	2%	1%	2%	10%	10%	10%
Sulfate	11%	9%	11%	12%	9%	12%	5%	10%	5%
